# Association of *CD247* Polymorphisms with Rheumatoid Arthritis: A Replication Study and a Meta-Analysis

**DOI:** 10.1371/journal.pone.0068295

**Published:** 2013-07-05

**Authors:** María Teruel, Cushla McKinney, Alejandro Balsa, Dora Pascual-Salcedo, Luis Rodriguez-Rodriguez, Ana M. Ortiz, Carmen Gómez-Vaquero, Miguel A. González-Gay, Malcolm Smith, Torsten Witte, Tony Merriman, Benedicte A. Lie, Javier Martin

**Affiliations:** 1 Instituto de Parasitología y Biomedicina ‘López-Neyra’, Granada, Spain; 2 Department of Biochemistry, University of Otago, Dunedin, New Zeland; 3 Rheumatology Service, Hospital Universitario La Paz, Madrid, Spain; 4 Rheumatology Service, Hospital Clínico San Carlos, Madrid, Spain; 5 Rheumatology Service, Hospital Universitario La Princesa, Instituto de Investigación Sanitaria La Princesa, Madrid, Spain; 6 Rheumatology Service, Hospital Universitari Bellvitge, Barcelona, Spain; 7 Rheumatology Division, Hospital Universitario Marqués de Valdecilla, Santander, Spain; 8 Department of Medicine, Flinders Medical Centre and Repatriation General Hospital, Adelaide, Australia; 9 Clinic for Immunology and Rheumatology Medical School, Hannover, Germany; 10 Department of Medical Genetics, University of Oslo and Oslo University Hospital, Oslo, Norway; The Children's Hospital of Philadelphia, United States of America

## Abstract

Given the role of CD247 in the response of the T cells, its entailment in autoimmune diseases and in order to better clarify the role of this gene in RA susceptibility, we aimed to analyze *CD247* gene variants previously associated with other autoimmune diseases (rs1052237, rs2056626 and rs864537) in a large independent European Caucasian population. However, no evidence of association was found for the analyzed *CD247* single-nucleotide polymorphisms (SNPs) with RA and with the presence/absence of anti-cyclic citrullinated polypeptide. We performed a meta-analysis including previously published GWAS data from the rs864537 variant, revealing an overall genome-wide significant association between this *CD247* SNP and RA with anti-CCP (OR = 0.90, CI 95% = 0.87–0.93, P_overall_ = 2.1×10^−10^). Our results show for first time a GWAS-level association between this *CD247* polymorphism and RA risk.

## Introduction

The T-cell receptor T3 zeta chain (CD3ζ), also called CD247, is essential for assembly, surface expression and signaling cascade of the T-cell receptor-CD3 (TCR/CD3) complex. Abnormalities in this pathway can result in T-cell dysfunction and development of autoimmune disorders [1]. Regarding this, previous reports have demonstrated an association of *CD247* variants with systemic lupus erythematosus [2], [3] and systemic sclerosis [4], [5]. In the case of rheumatoid arthritis (RA), one meta-GWAS found no association between the *CD247* gene and RA at a genome-wide significance level [6]; however, a more recent meta-analysis of GWAS data from RA and celiac disease (CeD) identified *CD247* as a novel susceptibility *locus* for both diseases [7]. Taking into account the role of CD247 in the response of the T-cells, its entailment in autoimmune diseases and in order to better clarify the role of this gene in RA susceptibility, we aimed to analyze *CD247* gene variants in a large independent European Caucasian cohort.

## Materials and Methods

A large independent European Caucasian cohort containing cases from four different countries has been included in the present study. The individuals were recruited from hospitals of Spain (1,797 cases and 2,026 controls), Germany (326 cases and 379 controls), Norway (949 cases and 1,121 controls) and Australasian (488 cases and 508 controls). All cases were ascertained by the American College of Rheumatology criteria [8]. Briefly, the 75% of the patients were females, the 76% were rheumatoid factor (RF) positive and the 63% were anti-cyclic citrullinated polypeptide (anti-CCP) positive. Controls self- reported as not having RA.

All subjects provide written informed consent according to the declaration of Helsinki. The study was approved by the ethical committees from all the Spanish participating centers to Galicia, Madrid (Hospital Clínico San Carlos, Hospital La Paz and Hospital de La Princesa), Barcelona (Hospital Universitario Bellvitge) and Santander (Hospital Universitario Marqués de Valdecilla). In addition, the ethical committees of Medizinische Hochschule Hannover from Germany, the Regional Committees for Research Ethics in Eastern and Southern Norway and the Multi-region Ethics Committee and the Lower South Ethics Committee of New Zealand also approved the study.

Three *CD247* single-nucleotide polymorphisms (SNPs) previously associated with different autoimmune diseases including RA were selected: rs1052237 [2], [3], rs2056626 [5] and rs864537 [7], and genotyped using TaqMan assays. A total of 350 patients were genotyped twice to verify the genotyping consistency, showing 99% identical genotypes.

In order to increase the robustness of our analyses, we conducted a meta-analysis with previously published data. The searching of the articles was performed on MEDLINE and PUBMED electronic databases. Studies for the meta-analysis were selected if they met the following conditions: 1) RA patients fulfilled the ACR criteria of 1988; 2) data were collected in European Caucasian populations; 3) they included any of the three polymorphisms selected in the present study. Given these considerations, only two articles were included in our study [6], [7].

The Hardy-Weinberg equilibrium (HWE) was tested by the means of χ^2^ or Fisher’s exact test when necessary. The case-control association study was performed by 2×2 contingency table with χ^2^ to obtain *P*-values, odds ratios (OR) and 95% confidence intervals (CI). Combined ORs were calculated according to a fixed-effects model (Mantel–Haenszel meta-analysis) and the heterogeneity of ORs among all populations was calculated by Breslow-Day test. P values lower than 0.0007 were regarded as significant after applying the Bonferroni correction. Statistical analyses were carried out with Plink v1.7 [9] (see URL: http://pngu.mgh.harvard.edu/~purcell/plink/) and StatsDirect software v2.4.6.

The estimation of the power of the study to detect an effect of a polymorphism in disease susceptibility was performed using the the CaTS Power Calculator software [10] (see URL: http://www.sph.umich.edu/csg/abecasis/CaTS).

## Results and Discussion

The estimated power of the study was 61% for rs1052237 and 82% for rs2056626 and rs864537 to detect ORs = 1.10 at the 5% significant level. The genotyping call-rate success was 98.3% in cases and 99.0% in controls for rs1052231, 97.9% in cases and 96.9% in controls for rs864537 and 97.6% in cases and 96.4% in controls for rs2056626. Genotype frequencies were in HWE in cases and controls. The distribution of genotypic and allelic frequencies for each population is shown in Table S1. We did not observe any significant association after the multiple testing correction was applied. However, the meta-analysis of the four cohorts using the Mantel-Hanzel method under fixed effects model showed no evidence of association of the three *CD247* SNPs and RA (rs1052231: OR = 1.02, CI 95% = 0.94–1.20, p = 0.595; rs864537: OR = 0.97, CI 95% = 0.91–1.03, p = 0.330; rs2056626: OR = 0.97, CI 95% = 0.91–1.04, p = 0.348) (see *Table 1*). The combinability test according to the Breslow-Day method showed no significant heterogeneity among the ORs of the different cohorts. Moreover, no evidence of association was observed after stratification for RF and anti-CCP status (*Table 1* *and Supplementary* *Table 1*).

**Table 1:** Combined analysis of *CD247* polymorphisms according to disease and serological status.

Samples					Minor allele (%)	Allele test*		
Sets	N	Genotype (%)				P-value	OR [95% CI]	P_BD_
*rs1052231*		*T/T*	*T/A*	*A/A*	*A*			
Controls	3994	69.1	27.6	2.9	16.7			
RA	3499	69.1	27.7	3.2	17.0	0.595	1.02 [0.94–1.20]	0.117
RA RF +	2094	68.5	28.2	3.3	17.4	0.463	1.04 [0.94–1.15]	0.080
RA RF −	991	69.1	28.0	2.9	16.9	0.792	1.02 [0.89–1.16]	0.506
RA anti-CCP +	1702	69.2	27.8	3.0	16.9	0.852	0.99 [0.88–1.10]	0.142
RA anti-CCP −	1013	68.2	29.2	2.6	17.2	0.451	0.95 [0.83–1.08]	0.192
*rs864537*		*A/A*	*A/G*	*G/G*	*G*			
Controls	3910	38.3	46.8	14.9	38.3			
RA	3485	39.0	46.8	14.2	37.6	0.33	0.97 [0.91–1.03]	0.648
RA RF +	2087	39.5	47.1	13.4	37.8	0.060	0.93 [0.86–1.00]	0.509
RA RF −	982	37.8	46.6	15.6	38.4	0.406	1.04 [0.94–1.56]	0.931
RA anti-CCP +	1699	38.8	47.5	13.7	37.5	0.457	0.97 [0.89–1.05]	0.482
RA anti-CCP −	1011	40.5	45.5	14.0	36.8	0.309	0.95 [0.86–1.05]	0.844
*rs2056626*		*T/T*	*T/G*	*G/G*	*G*			
Controls	3888	38.6	46.6	14.8	38.1			
RA	3473	39.2	46.8	14.0	37.4	0.348	0.97 [0.91–1.04]	0.051
RA RF +	2082	39.9	45.9	14.3	37.9	0.294	0.96 [0.87–1.04]	0.031
RA RF −	979	38.4	47.3	14.3	38.1	0.803	1.01 [0.91–1.12]	0.545
RA anti-CCP +	1683	40.6	45.1	14.3	36.9	0.323	0.96 [0.88–1.04]	0.031
RA anti-CCP −	1005	39.4	47.2	13.4	37.0	0.712	0.98 [0.89–1.09]	0.956

Controls are used as reference for all comparisons.

* Allelic Mantel-Haenszel fixed effects model.

RA, rheumatoid arthritis; RF, rheumatoid factor; anti-CCP, anti-cyclic citrullinated peptide; +, positive; −, negative; P_BD_, Breslow-Day P-Value.

**Figure 1:**
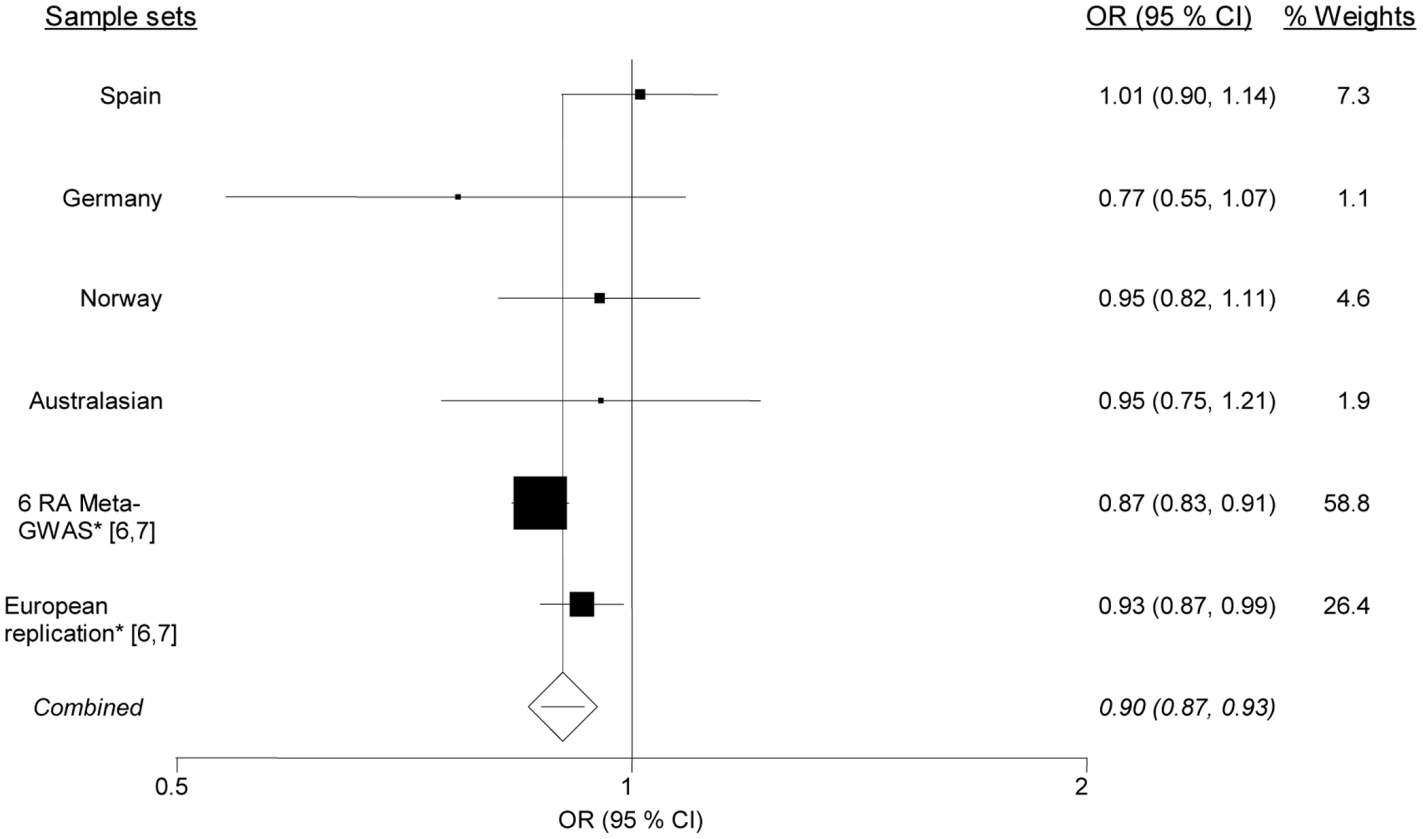
Forest plots of the *CD247 rs864537* variant in RA patients and healthy subjects including GWAS data from Stahl *et al.* [6] and Zhernakova et al. [7]. The combined analysis included a total of 10,763 RA patients with anti-CPP and 28,879 controls. There was no evidence of heterogeneity between sample sets (Breslow-Day test for heterogeneity p = 0.12).

In addition to these analyses, we performed a meta-analysis including published GWAS data for rs864537 [6], [7]. Considering that these studies included auto-antibody-positive RA individuals, we performed the combined analysis including only RA patients with anti-CCP. The data of these GWASs together with our combined data sets revealed an overall genome-wide significant association between rs864537 and RA with anti-CCP (OR = 0.90, Ci 95% = 0.87–0.93, P_overall_ = 2.1×10^−10^) (Figure 1).

The present study represents a good example of how increasing the study cohort may be necessary to detect genetic associations of weak effect size at GWAS level. In this regard, the study performed by Stahl *et al*. detected no association of *CD247* with RA at GWAS level [6], and the *CD247* association only reached genome-wide significance level when data of two autoimmune diseases, RA and CeD, were combined [7]. Therefore, the current study described for first time at GWAS-level association of *CD247* and RA risk using a meta-analysis of a large European Caucasian case-control sample set.

A reduced density of synovial T-cell surface CD3ζ has been observed in RA patients [11], [12], [13], suggesting a decrease in TCR signaling, thus favoring positive selection of autoreactive T effector cells in the thymus [14]. It is unclear what is the cause of the low expression of CD3ζ in synovial T cells and several possible mechanisms should be considered [1], [11], [12], [13]. Regarding this, splicing variants of *CD247* have been demonstrated to lead to downregulated protein expression in SLE patients [1], [15]. It is interesting to note that the risk variant of *CD247* rs864537 has also been correlated with *cis* gene expression of *CD247* [7]. Further functional studies should shed more light on the role of this genetic variant in the susceptibility to RA.

## Supporting Information

Table S1 (DOC)

Checklist S1 (DOC)

Diagram S1 (DOC)
