# Supplementary material for: Association of CD247 Polymorphisms with Rheumatoid Arthritis: A Replication Study and a Meta-Analysis
Source: PLoS One. 2013 Jul 5;8(7):e68295. doi: 10.1371/journal.pone.0068295 (PMC3702579; doi:10.1371/journal.pone.0068295)
Supplement: Table S1 — (DOC) [file pone.0068295.s001.doc]

**Supplementary Table 1**. Distribution of the genetic frequencies of *CD247* polymorphisms in five populations.

|  | Samples |  |  |  |  | Minor | Allele test* | |
| --- | --- | --- | --- | --- | --- | --- | --- | --- |
|  | Sets | N | Genotype (%) | | | allele (%) | P-value‡ | OR [95% CI] |
| *rs1052231* |  |  | *T/T* | *T/A* | *A/A* | *A* |  |  |
| Spain | Controls | 1999 | 69.1 | 28.2 | 2.7 | 16.8 |  |  |
|  | RA | 1756 | 66.6 | 29.6 | 3.8 | 18.6 | 0.043 | 1.13 [1.00-1.27] |
|  | RA RF + | 1117 | 66.0 | 30.0 | 4.0 | 19.0 | 0.028 | 1.16 [1.02-1.33] |
|  | RA RF - | 472 | 67.8 | 29.0 | 3.2 | 17.7 | 0.516 | 1.06 [0.88-1.28] |
|  | RA anti-CCP + | 860 | 66.5 | 29.8 | 3.7 | 18.6 | 0.100 | 1.13 [0.98-1.31] |
|  | RA anti-CCP - | 550 | 64.5 | 32.6 | 2.9 | 19.2 | 0.065 | 1.18 [0.99-1.39] |
| Germany | Controls | 375 | 66.4 | 30.7 | 2.9 | 18.3 |  |  |
|  | RA | 313 | 68.1 | 28.1 | 3.8 | 17.9 | 0.857 | 0.98 [0.74-1.29] |
|  | RA RF + | 130 | 65.4 | 31.5 | 3.1 | 18.9 | 0.835 | 1.04 [0.72-1.49] |
|  | RA RF - | 31 | 80.6 | 16.2 | 3.2 | 11.3 | 0.167 | 0.57 [0.25-1.28] |
|  | RA anti-CCP + | 101 | 73.3 | 24.7 | 2.0 | 14.4 | 0.194 | 0.75 [0.49-1.16] |
|  | RA anti-CCP - | 32 | 62.5 | 31.2 | 6.3 | 21.9 | 0.476 | 1.25 [0.67-2.33] |
| Norway | Controls | 1116 | 70.2 | 26.4 | 3.4 | 16.6 |  |  |
|  | RA | 944 | 71.9 | 25.3 | 2.8 | 15.4 | 0.293 | 0.91 [0.77-1.08] |
|  | RA RF + | 478 | 73.4 | 23.5 | 3.1 | 14.9 | 0.213 | 0.88 [0.71-1.08] |
|  | RA RF - | 407 | 68.8 | 28.5 | 2.7 | 17.0 | 0.828 | 1.02 [0.83-1.27] |
|  | RA anti-CCP + | 546 | 71.8 | 25.5 | 2.7 | 15.5 | 0.400 | 0.92 [0.75-1.12] |
|  | RA anti-CCP - | 337 | 72.1 | 25.8 | 2.1 | 15.0 | 0.313 | 0.88 [0.70-1.12] |
| Australasian | Controls | 504 | 74.2 | 24.4 | 1.4 | 13.6 |  |  |
|  | RA | 486 | 71.6 | 26.5 | 1.9 | 15.1 | 0.331 | 1.13 [0.88-1.46] |
|  | RA RF + | 369 | 71.0 | 27.6 | 1.4 | 15.2 | 0.350 | 1.14 [0.87-1.49] |
|  | RA RF - | 81 | 74.1 | 23.4 | 2.5 | 14.2 | 0.835 | 1.05 [0.65-1.69] |
|  | RA anti-CCP + | 195 | 71.3 | 27.7 | 1.0 | 14.9 | 0.535 | 1.11 [0.80-1.55] |
|  | RA anti-CCP - | 94 | 71.3 | 26.6 | 2.1 | 15.4 | 0.504 | 1.16 [0.75-1.79] |
| *rs864537* |  |  | *A/A* | *A/G* | *G/G* | *G* |  |  |
| Spain | Controls | 1923 | 40.0 | 44.3 | 15.7 | 37.8 |  |  |
|  | RA | 1743 | 38.5 | 47.3 | 14.2 | 37.9 | 0.976 | 1.00 [0.91-1.10] |
|  | RA RF + | 1111 | 39.1 | 47.2 | 13.7 | 37.3 | 0.686 | 0.98 [0.88-1.09] |
|  | RA RF - | 462 | 35.7 | 49.8 | 14.5 | 39.4 | 0.380 | 1.07 [0.92-1.24] |
|  | RA anti-CCP + | 858 | 38.1 | 47.6 | 14.3 | 38.1 | 0.842 | 1.01 [0.90-1.14] |
|  | RA anti-CCP - | 545 | 39.6 | 47.9 | 12.5 | 36.4 | 0.396 | 0.94 [0.82-1.08] |
| Germany | Controls | 362 | 39.8 | 43.3 | 16.9 | 38.5 |  |  |
|  | RA | 305 | 40.3 | 48.2 | 11.5 | 35.6 | 0.265 | 0.88 [0.70-1.10] |
|  | RA RF + | 126 | 44.4 | 43.7 | 11.9 | 33.7 | 0.174 | 0.81 [0.60-1.10] |
|  | RA RF - | 28 | 42.9 | 42.8 | 14.3 | 35.7 | 0.676 | 0.89 [0.50-1.56] |
|  | RA anti-CCP + | 97 | 46.4 | 42.3 | 11.3 | 32.5 | 0.121 | 0.77 [0.55-1.07] |
|  | RA anti-CCP - | 32 | 43.8 | 37.4 | 18.8 | 37.5 | 0.870 | 0.96 [0.56-1.62] |
| Norway | Controls | 1118 | 38.6 | 49.3 | 12.1 | 36.7 |  |  |
|  | RA | 949 | 41.6 | 44.9 | 13.5 | 35.9 | 0.601 | 0.97 [0.85-1.10] |
|  | RA RF + | 480 | 44.0 | 45.0 | 11.0 | 33.5 | 0.086 | 0.87 [0.74-1.02] |
|  | RA RF - | 410 | 40.5 | 43.9 | 15.6 | 37.6 | 0.669 | 1.04 [0.88-1.22] |
|  | RA anti-CCP + | 548 | 41.1 | 46.7 | 12.2 | 35.6 | 0.523 | 0.95 [0.82-1.11] |
|  | RA anti-CCP - | 340 | 42.1 | 42.6 | 15.3 | 36.6 | 0.962 | 1.00 [0.83-1.19] |
| Australasian | Controls | 507 | 30.0 | 53.0 | 17.0 | 43.5 |  |  |
|  | RA | 488 | 34.8 | 48.0 | 17.2 | 41.2 | 0.299 | 0.91 [0.76-1.09] |
|  | RA RF + | 370 | 33.5 | 50.3 | 16.2 | 41.4 | 0.371 | 0.92 [0.76-1.11] |
|  | RA RF - | 82 | 34.1 | 43.9 | 22.0 | 43.9 | 0.922 | 1.02 [0.73-1.42] |
|  | RA anti-CCP + | 196 | 31.6 | 52.1 | 16.3 | 42.4 | 0.698 | 0.95 [0.75-1.21] |
|  | RA anti-CCP - | 94 | 38.3 | 44.7 | 17.0 | 39.4 | 0.293 | 0.84 [0.61-1.16] |
| *rs2056626* |  |  | *T/T* | *T/G* | *G/G* | *G* |  |  |
| Spain | Controls | 1970 | 43.0 | 43.3 | 13.7 | 35.3 |  |  |
|  | RA | 1768 | 40.2 | 47.1 | 12.7 | 36.3 | 0.404 | 1.04 [0.95-1.15] |
|  | RA RF + | 1126 | 40.2 | 46.9 | 12.9 | 36.3 | 0.433 | 1.04 [0.94-1.16] |
|  | RA RF - | 473 | 39.7 | 47.6 | 12.7 | 36.5 | 0.511 | 1.05 [0.91-1.22] |
|  | RA anti-CCP + | 864 | 40.4 | 46.5 | 13.1 | 36.3 | 0.464 | 1.05 [0.93-1.18] |
|  | RA anti-CCP - | 554 | 41.0 | 47.1 | 11.9 | 35.5 | 0.932 | 1.01 [0.88-1.16] |
| Germany | Controls | 373 | 30.3 | 52.0 | 17.7 | 43.7 |  |  |
|  | RA | 315 | 39.0 | 47.7 | 13.3 | 37.1 | 0.014 | 0.76 [0.61-0.95] |
|  | RA RF + | 133 | 45.1 | 38.4 | 16.5 | 35.7 | 0.023 | 0.72 [0.54-0.96] |
|  | RA RF - | 31 | 41.9 | 45.2 | 12.9 | 35.5 | 0.209 | 0.71 [0.41-1.22] |
|  | RA anti-CCP + | 102 | 47.1 | 38.2 | 14.7 | 33.8 | 0.011 | 0.66 [0.48-0.91] |
|  | RA anti-CCP - | 33 | 36.4 | 42.4 | 21.2 | 42.4 | 0.841 | 0.95 [0.57-1.58] |
| Norway | Controls | 1037 | 37.0 | 48.5 | 14.5 | 38.7 |  |  |
|  | RA | 903 | 41.0 | 44.8 | 14.2 | 36.6 | 0.175 | 0.91 [0.80-1.04] |
|  | RA RF + | 454 | 43.4 | 43.6 | 13.0 | 34.8 | 0.042 | 0.84 [0.72-0.99] |
|  | RA RF - | 393 | 38.4 | 46.3 | 15.3 | 38.4 | 0.885 | 0.99 [0.83-1.17] |
|  | RA anti-CCP + | 522 | 42.3 | 44.1 | 13.6 | 35.6 | 0.093 | 0.88 [0.75-1.02] |
|  | RA anti-CCP - | 324 | 39.2 | 46.0 | 14.8 | 37.8 | 0.678 | 0.96 [0.80-1.15] |
| Australasian | Controls | 508 | 30.9 | 51.4 | 17.7 | 43.4 |  |  |
|  | RA | 487 | 32.9 | 48.2 | 18.9 | 43.0 | 0.862 | 0.98 [0.82-1.18] |
|  | RA RF + | 369 | 32.5 | 48.3 | 19.2 | 43.4 | 0.985 | 1.00 [0.82-1.21] |
|  | RA RF - | 82 | 29.3 | 51.2 | 19.5 | 45.1 | 0.681 | 1.07 [0.77-1.49] |
|  | RA anti-CCP + | 195 | 33.3 | 45.2 | 21.5 | 44.1 | 0.814 | 1.03 [0.81-1.30] |
|  | RA anti-CCP - | 94 | 31.9 | 53.2 | 14.9 | 41.5 | 0.626 | 0.92 [0.67-1.27] |

Controls are used as reference for all comparisons.

* Allele test performed by χ ^2^ in 2x2 contingency tables.

‡P values lower than 0.0007 were regarded as significant after applying the Bonferroni correction.

RA, rheumatoid arthritis; RF, rheumatoid factor; anti-CCP, anti-cyclic citrullinated peptide; +, positive; -, negative.
