# Supplementary material for: Association of CD247 Polymorphisms with Rheumatoid Arthritis: A Replication Study and a Meta-Analysis
Source: PLoS One. 2013 Jul 5;8(7):e68295. doi: 10.1371/journal.pone.0068295 (PMC3702579; doi:10.1371/journal.pone.0068295)
Supplement: Diagram S1 — (DOC) [file pone.0068295.s003.doc]

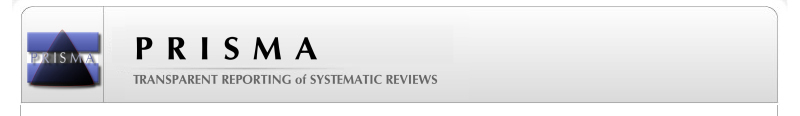
**PRISMA 2009 Flow Diagram**

**Screening**

**Included**

**Eligibility**

**Identification**

Records identified through database searching
(n =7)

Additional records identified through other sources
(n = )

Records after duplicates removed
(n =7)

Records screened
(n =7)

Records excluded
(n = )

Full-text articles assessed for eligibility
(n =2)

Full-text articles excluded, with reasons
(n =5)

Studies included in qualitative synthesis
(n = 2)

Studies included in quantitative synthesis (meta-analysis)
(n =2)
